# The Effect of Transcranial Alternating Current Stimulation With Cognitive Training on Executive Brain Function in Individuals With Dementia: Protocol for a Crossover Randomized Controlled Trial

**DOI:** 10.2196/37282

**Published:** 2022-04-27

**Authors:** Natasha Jacobson, Brian Lithgow, Mohammad Jafari Jozani, Zahra Moussavi

**Affiliations:** 1 Department of Electrical and Computer Engineering University of Manitoba Winnipeg, MB Canada; 2 Monash Alfred Psychiatry Research Centre Monash University Central Clinical School Melbourne Australia; 3 Department of Statistics University of Manitoba Winnipeg, MB Canada

**Keywords:** transcranial alternating current stimulation, Alzheimer disease, cognitive impairment, double blind, treatment, placebo-controlled, randomized, crossover, dementia, cognitive

## Abstract

**Background:**

Although memory and cognitive declines are associated with normal brain aging, they may also be precursors to dementia.

**Objective:**

We aim to offer a novel approach to prevent or slow the progress of neurodegenerative dementia, or plausibly, improve the cognitive functions of individuals with dementia.

**Methods:**

We will recruit and enroll 75 participants (older than 50 years old with either mild cognitive impairment or probable early or moderate dementia) for this double-blind randomized controlled study to estimate the efficacy of active transcranial alternating current stimulation with cognitive treatment (in comparison with sham transcranial alternating current stimulation). This will be a crossover study; a cycle consists of sham or active treatment for a period of 4 weeks (5 days per week, in two 30-minute sessions with a half-hour break in between), and participants are randomized into 2 groups, with stratification by age, sex, and cognitive level (measured with the Montreal Cognitive Assessment). Outcomes will be assessed before and after each treatment cycle. The primary outcomes are changes in Wechsler Memory Scale Older Adult Battery and Alzheimer Disease Assessment Scale scores. Secondary outcomes are changes in performance on tests of frontal lobe functioning (verbal fluency), neuropsychiatric symptoms (Neuropsychiatric Inventory Questionnaire), mood changes (Montgomery-Åsberg Depression Rating Scale), and short-term recall (visual 1-back task). Exploratory outcome measures will also be assessed: static and dynamic vestibular response using electrovestibulography, neuronal changes using functional near-infrared spectroscopy, and change in spatial orientation using virtual reality navigation.

**Results:**

As of February 10, 2022, the study is ongoing: 7 patients have been screened, and all were deemed eligible for and enrolled in the study; 4 participants have completed baseline assessments.

**Conclusions:**

We anticipate that transcranial alternating current stimulation will be a well-tolerated treatment, with no serious side effects and with considerable short- and long-term cognitive improvements.

**Trial Registration:**

Clinicaltrials.gov NCT05203523; https://clinicaltrials.gov/show/NCT05203523

**International Registered Report Identifier (IRRID):**

DERR1-10.2196/37282

## Introduction

### Background

Due to medical advancements and healthier lifestyles, lifespans are increasing. However, as longevity increases, cognitive abilities such as executive function, memory, reasoning, and processing speed deteriorate [1]. While deterioration may be part of normal aging [2], declines in cognitive function or associative and spatial memories can also be precursors to dementia [3]. There is currently no cure for dementia, however, there is hope that the onset of the disease can be delayed, or its progression slowed, by living a brain-healthy lifestyle. This hope is based on brain neuroplasticity, as well as individuals’ cognitive reserves [4]. It has been suggested that constructing a cognitive “reserve capacity” [5] can help seniors to maintain cognitive function, which was later supported by neuroimaging studies that showed increased contralateral hemispheric activity in right frontal regions for both working memory [6] and episodic memory [7] in aging populations.

Recently, many interventions and studies [8-10] have reportedly demonstrated cognitive improvement in older adults from cognitive training (brain exercises or brain games), if they are used frequently and regularly; however, cognitive improvements were largely observed in the same tasks, with minimal far-transfer effects being observed. A 2018 study with 72 participants (20 to 62 years) showed evidence against transferable gains from cognitive function training that consisted of spatial training and working memory tasks [11]. However, in almost all studies that have considered the effect of brain exercises on the cognitive function of adults and older adults, participants performed their exercises without a trainer [8,11]. Conversely, significant cognitive improvement in individuals with mild or moderate dementia has been demonstrated when they participated in daily brain exercises in a regimented, structured learning environment with the help of a tutor, and improvements transferred to their daily life beyond the practiced exercises as the outcome measures were independent of the practiced tasks [9,12-14].

The application of transcranial alternating current stimulation—an external oscillating electrical field that induces cortical activity—either in addition to or independent of brain exercises, on cognition has been explored. It is a relatively inexpensive, easy to administer, and safe tool for noninvasive brain stimulation. Transcranial alternating current stimulation has been demonstrated to both modulate and entrain the ongoing network oscillations in a frequency-specific manner [15], by using appropriate stimulation parameters (ie, frequency, intensity, duration, and anatomical location) to manipulate the phase, the rhythm, and the power of neural oscillations, through in vitro and in vivo experiments [16]. Because it operates in a frequency- and phase-specific manner, which offers the possibility to demonstrate causal relations between oscillations and behavior [17,18], interest in transcranial alternating current stimulation has significantly increased in the past decade. Furthermore, the therapeutic potential of transcranial alternating current stimulation has led many researchers to study its applicability as a treatment option for numerous neurological and psychological disorders [14,18,19]. However, there has been little investigation into the potential effect of transcranial alternating current stimulation on older adults with cognitive impairments associated with dementia. Moreover, much of the research on transcranial alternating current stimulation involves testing participants’ cognitive performance during or immediately after stimulation sessions.

In a recent pilot study [14], we demonstrated that the addition of transcranial alternating current stimulation to a cognitive training program improved participants’ working memory, and improvements remained for a longer period after the intervention; however, there were several shortcomings in this study. Aside from having a limited sample size, the main shortcoming was that placebo effect was not analyzed. We aim to address shortcomings by employing a better study design to investigate the efficacy of combined transcranial alternating current stimulation and cognitive training on the dementia population (Clinicaltrials.gov NCT05203523).

### Objective

We aim to investigate the effect of transcranial alternating current stimulation when paired with simultaneous cognitive training on the cognitive status of the dementia population as well as the predictability of participant responses to active transcranial alternating current stimulation at baseline.

### Hypotheses

We hypothesize that (1) better cognitive performance will be evident for patients’ active treatment periods compared with those from the sham periods; (2) in both groups, statistically significant improvement in cognitive performance will be evident immediately postintervention compared with baseline, and (3) a statistically significant difference in cognitive improvement will be found between the active and sham transcranial alternating current stimulation groups.

## Methods

### Experimental Design

This is a randomized, crossover, double-blind, placebo-controlled study. Participants with cognitive impairments are recruited at 1 of 2 sites (Manitoba and Alberta) and randomized into 2 groups, with stratification by age, sex, and cognitive level (measured with Montreal Cognitive Assessment [20]) for randomization. Group 1 participants receive active transcranial alternating current stimulation simultaneously with cognitive exercises. Group 2 participants receive sham transcranial alternating current stimulation simultaneously with cognitive exercises. Participants who cannot tolerate the application of transcranial alternating current stimulation and focus on the cognitive exercises at the same time are enrolled in a third group, in which they only receive cognitive exercises but otherwise follow the same protocol as those in group 1 and group 2. The outcomes of group 3 will be analyzed separately. Standard cognitive assessments are performed before and after treatment, as well as at a scheduled follow-up visit 3 months postintervention. Both participants and assessors remain blind to the type of treatment (active versus placebo) until the end of the study.

### Recruitment

Approximately 75 patients with either mild cognitive impairment or probable early or moderate dementia, excluding Parkinsonian dementia, as confirmed by their treating physician will be recruited and tested over the course of this study. Participants are recruited from volunteers or doctor referrals.

Participant eligibility is confirmed in-person using the Montreal Cognitive Assessment, to assess the severity of dementia, and the Montgomery-Åsberg Depression Rating Scale, to assess for comorbid depression. Inclusion criteria are (all must be met): age between 50 and 95 years old; Montreal Cognitive Assessment score between 5 and 24; and the ability to read, write, and speak English fluently.

The exclusion criteria are diagnosis of Parkinson, Parkinsonian dementia, Huntington disease, speech-significant aphasia, intellectual disability, major depression or anxiety, bipolar disorder, schizophrenia, or any other major mood disorder; a history of epileptic seizures or epilepsy; inability to adequately communicate in English; vision or hearing that is sufficiently impaired to affect performance in cognitive tests; current substance abuse disorder; current participation in another therapeutic study for dementia; or a plan to change medication during the study period.

After the initial screening process and before randomization (Proc Plan, version 9.4; SAS Institute), participant tolerance to transcranial alternating current stimulation is tested with a 1-minute application as they focus on a cognitive exercise. If the participant cannot tolerate the application of transcranial alternating current stimulation, they are offered a chance to participate in the study as part of group 3. If they can tolerate the transcranial alternating current stimulation, they are randomized into either group 1 or group 2.

Prior to study participation, all patients and their primary caregivers are required to sign an informed consent form approved by the ethics board of each site of the study.

### Randomization

Using stratified block randomization (block size of 6) [21], participants are assigned into 1 of the 2 equal-size age-, sex-, and severity-matched groups: group 1 and group 2 (Table 1). Balanced randomization is applied within each block, such that 3 participants receive the active transcranial alternating current stimulation treatment and the rest receive the sham treatment. This randomization procedure is used to avoid bias in statistical analyses comparing the 2 groups.

**Table 1:** Stratified blocks using 2 levels for 3 factors.

Block number	Factor		
	Age (years)	Sex	Severity (Montreal Cognitive Assessment score)
1	≥70	Female	≥18
2	≥70	Female	<18
3	≥70	Male	≥18
4	≥70	Male	<18
5	<70	Female	≥18
6	<70	Female	<18
7	<70	Male	≥18
8	<70	Male	<18

### Treatment Protocol

**Figure 1:**
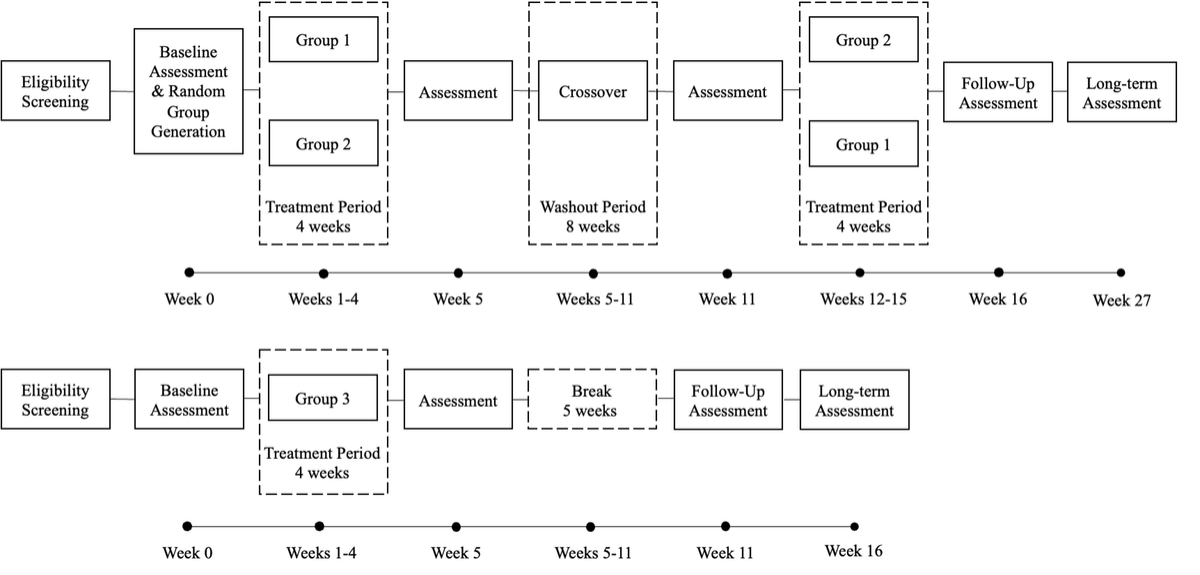
Study flowchart for groups 1 to 3.

Training for all groups occurs in-person over the course of 4 weeks, 5 days per week (excluding weekends); there is an 8-week wash-out period prior to group crossover. On each day, participants attend two 30-minute training sessions using an app (MindTriggers) with a 30-minute break in between. Assessments occur at baseline (week 0), postintervention (weeks 5 and 11), at a follow-up (week 16), and at a long-term follow-up (week 27). Assessment at week 11 is considered to be baseline for the second cycle. No crossover occurs for participants assigned to group 3 (Figure 1).

During a learning or problem-solving task, it has been shown that gamma band waves (brain waves faster than 30 Hz) are generated [22]. Previous work has suggested the potential of gamma band stimulation to improve cognitive performance in dementia patients [23], improve working memory [24], and increase attentiveness [25]. Furthermore, the reduction of intracerebral tau protein burden has been proposed as an added benefit of gamma band stimulation [26]. Transcranial alternating current stimulation is one such means of gamma band stimulation. Recent work [14] further supported cognitive improvement in older adults with more sustained improvement in participants who underwent transcranial alternating current stimulation. However, the use of small sample sizes in all studies [14,23-26] limited the conclusions that could be drawn. Thus, given the benefits of gamma band stimulation, transcranial alternating current stimulation is applied at a frequency of 40 Hz with a current density of 0.04 mA/cm^2^.

Therefore, participants in group 1 and group 2 receive transcranial alternating current stimulation treatment simultaneously with cognitive exercises. The transcranial alternating current stimulation treatment (Model 2001; Soterix Medication) is applied with a sinusoidal waveform at 40 Hz, with a current amplitude of –0.75 mA to 0.75 mA (1.5 mA peak-to-peak). Electrode placement is measured at the first training session, with the active electrode being placed over the left dorsolateral prefrontal cortex, and the reference electrode being placed on the contralateral supraorbital area.

To prevent unblinding, the auto-sham toggle on the transcranial alternating current stimulation machine is hidden from assessors with a physical barrier. The auto-sham sequence provides the same sensory experience as active transcranial alternating current stimulation by providing a ramp up and down to 1.5 mA at the start of treatment, and again after 10 minutes.

### Outcome Measures

At each assessment, 9 tests are conducted over 2 days due to time constraints. The first assessment day comprises the Wechsler Memory Scale Older Adult Battery [27], Montgomery-Åsberg Depression Rating Scale, a verbal fluency test for speech analysis, a visual 1-back task [28], virtual reality navigation [29], the Neuropsychiatric Inventory Questionnaire, and functional near-infrared spectroscopy. The second assessment day includes electrovestibulography and the Alzheimer Disease Assessment Scale (ADAS) cognitive subscale. An assessor is assigned to each participant and performs all 4 assessments with the assigned participant. Only the study participant and the research personnel designated to administer assessments are present during assessments.

The primary outcomes are the changes in patients’ scores from baseline in the Wechsler Memory Scale Older Adult Battery and ADAS cognitive subscale assessments. Alternate forms of the ADAS cognitive subscale word lists are employed at each visit to reduce possible practice effects. The secondary outcomes are change in 1-back, Neuropsychiatric Inventory Questionnaire, and Montgomery-Åsberg Depression Rating Scale performance. The 1-back test presents a sequence of random shapes and participants must recall whether a presented object is a repeat of the previous object [28]. Neuropsychiatric Inventory Questionnaire is used to assesses neuropsychiatric symptoms and caregiver burden. Finally, to establish the presence of confounding variables, namely mood disorders, Montgomery-Åsberg Depression Rating Scale is evaluated at each assessment. The secondary exploratory outcome measures use a series of physiological monitoring tools to examine physiological changes in study participants before and after the intervention: (1) virtual reality navigation test to assess spatial orientation [29], (2) electrovestibulography [30-32] to assess static and dynamic vestibular response, (3) functional near-infrared spectroscopy (OctaMonas) [33] to record prefrontal cortex brain activities, and (4) verbal fluency to evaluate acoustic and linguistic changes (Table 2). It should be noted that the exploratory assessments will only be conducted at the Manitoba site.

**Table 2:** Study outcome measures.

Outcome measures		Assessments
**Primary**		
	Wechsler Memory Scale Older Adult Battery	Older adult memory
	Alzheimer Disease Assessment Scale cognitive subscale	Cognitive dysfunction level in Alzheimer disease
**Secondary**		
	Visual 1-back task	Immediate recall
	Neuropsychiatric Inventory Questionnaire	Neuropsychiatric symptoms; caregiver burden
	Montgomery-Åsberg Depression Rating Scale	Mood disorders
**Exploratory**		
	Functional near-infrared spectroscopy	Functional brain activity
	Virtual reality navigation	Spatial orientation
	Electrovestibulography	Static and dynamic vestibular response
	Verbal fluency	Acoustic and linguistic changes

#### Virtual Reality Navigation as a Test for Spatial Orientation

Virtual reality navigation assists in the detection of cognitive impairment by measuring how people orient themselves in an unfamiliar environment [29]. The virtual reality navigation test has two stages of assessment, which are completed on a laptop: (1) target localization from outside a building, and (2) target location inside a building.

#### Electrovestibulography

Electrovestibulography is a noninvasive recording made from the vestibulo-acoustic system with no motion and with passive whole-body tilt [30-32]. For this study, measurements are performed before and after treatment in order to assess the ability of electrovestibulography to predict treatment outcomes.

#### Functional Near-Infrared Spectroscopy

Functional near-infrared spectroscopy is an optical technique that uses near-infrared light, which is capable of penetrating the scalp, skull, and other brain tissues to reach gray matter, in order to noninvasively monitor functional brain activity by measuring the flow of oxygenated and deoxygenated blood [33]. Its main benefits are portability, noninvasiveness, and relatively high temporal resolution (100 ms) [33].

### Safety Considerations

The transcranial alternating current stimulation procedure used in this study is of minimal risk, though discomfort may arise. Common adverse effects of transcranial alternating current stimulation include mild headache, facial twitches, itching, redness under the electrodes, and light flashes [18]. The application of transcranial alternating current stimulation should be stopped if facial twitches or light flashes are experienced by the participant. Participants are asked at each visit if they have experienced any adverse effects from the treatment. Participants’ self-assessment of any pain or discomfort from the treatment are also recorded at every visit. To match similar studies [34], participant transcranial alternating current stimulation sensations are rated on a scale from 1 to 5 at each session, with 1 indicating no sensations, 2 indicating mild sensations, 3 indicating moderate sensations, 4 indicating strong sensations, and 5 indicating very strong sensations.

Assessment technologies also incur minimal risk. Dizziness may occur during virtual reality navigation, the head band used in functional near-infrared spectroscopy may cause discomfort, and electrovestibulography can result in ear infection if the ears are not properly cleaned after the assessment. In the case of any unexpected issue, or pain and intolerance experienced by a participant, assessments will be halted and risks will be reassessed. All issues will be reported to the appropriate ethics board.

### Statistical Analysis

#### Sample Size Estimation

This study will have 2 equal-sized groups, and it is hypothesized that one group’s mean will be significantly different from the other's in one primary outcome measure. As such, the sample size for each group can be calculated [35]. The expected difference and standard deviation for Wechsler Memory Scale Older Adult Battery mean scores between the 2 groups were 20 and 38, respectively, based on the results from a pilot study [14], from which individuals with a Montreal Cognitive Assessment score <5 were removed. With a test power of 80% and significance of 5%, the minimum sample size was estimated as 58 per group. However, due to the study’s crossover design, this number was halved. Allowing for a 5% dropout rate, and 20% transcranial alternating current stimulation intolerance rate [14], a total of 75 participants should be enrolled across all 3 groups. Of the total participants, approximately 30 are expected to be recruited at the Alberta study site, and the remaining participants are expected to be recruited at the Manitoba study site.

### Analysis

Both parametric and nonparametric statistical techniques will be used. Analysis of the repeated measures crossover data will be performed using fixed and random effect models to investigate different sources of variations in the data set such as period effect, direct treatment effect, and carryover treatment effects [36]. If raw data are found to not be normally distributed, the Box-Cox transformation will be employed to satisfy the normality assumption needed to perform the analysis [37].

In addition, the differences between study cycle data of the basic crossover design will be analyzed (that is, the differences between active and sham transcranial alternating current stimulation results). Study cycle data differences are used to transform the repeated measurements crossover design to a completely randomized repeated measurements design. With the transformed repeated measurements completely randomized design, analysis is completed by a univariate split-plot analysis of variance or multivariate (over time) *F* tests [38]. Should normality be in question due to the relatively small sample size, an alternative approach will be used based on nonparametric tests using ranks [39]. Nonparametric tests will include Wilcoxon rank sum and permutation tests to investigate hypotheses 1 and 2 and examine the absence of carryover effects in the study [39].

To have a better understanding of the data set, baseline measurements will be used to perform analysis of covariance. As such, the baseline crossover differences of each outcome measure (Table 2) are regarded as independent explanatory variables and regression of the basic estimators is calculated on said variables [36].

The abovementioned statistical analysis will be applied to the secondary outcome measures, as well.

### Ethics

Only participants able to give informed consent are recruited into the study. However, because the study population contains those with memory problems, a family member (or legal guardian) will be required to accompany the patient to the initial interview and sign the consent form for all participants.

Should a patient with Alzheimer disease be unable to provide informed consent, the person’s guardian, or an authority or other person having that responsibility at law, is required to provide consent to participate in the research on the individual’s behalf. Patients are advised that their participation is voluntary and that they are free to withdraw from the study at any stage.

Study data (participant medical and demographic data, treatment records, and assessment results) are maintained on a password-protected database accessible only to active research team members. All participants are assigned code numbers to ensure anonymity, with study files referencing participant code only. Regular backups of the database are performed and stored on a secure server. Identifying information (name, phone number, address) is not stored on the same system as study data and remains accessible only to staff members who need to contact participants.

Ethical approval for this study was received from the Biomedical Ethics Research Board at the University of Manitoba prior to participant recruitment at the Manitoba study location (HS25171 [B2021:089]). All participants or their legal guardians provide informed consent during the study.

## Results

As of February 10, 2022, the study is ongoing: 7 patients have been screened, and all were deemed eligible and enrolled in the study; 4 have completed baseline assessments.

## Discussion

This study’s design addresses both the short- and long-term effects of transcranial alternating current stimulation in a large study sample, by making comparisons with a sham treatment. Due to the COVID-19 pandemic, recruitment and enrollment slowed as a result of lockdowns imposed by the universities and health authorities.

The major limitation of the protocol is the lack of distinction between transcranial alternating current stimulation effects and cognitive training. A pilot study [14] showed cognitive improvement in patients with and without active transcranial alternating current stimulation treatment due to the combined effect of personal cognitive training. Thus, the independent effects of active transcranial alternating current stimulation treatment in comparison to sham transcranial alternating current stimulation are not discernible using this protocol. However, given the older age of study participants and often rapid degradation of cognitive function in dementia patients, it is the conscious effort of the research team to support brain stimulation even during sham treatments despite its confounding effect on results.

The study has been progressing as expected. Participants have found transcranial alternating current stimulation treatment to be agreeable and have adhered to outset eligibility criteria. Minor side effects, including fatigue and dizziness, have been appropriately addressed and managed during treatment.

## Appendix

Multimedia Appendix 1 Peer-review reports (1) from Mitacs Accelerate - Funded Internship Projects (Canada).

Multimedia Appendix 2 Peer-review reports (2) from Mitacs Accelerate - Funded Internship Projects (Canada).

Multimedia Appendix 3 CONSORT-eHEALTH checklist (V 1.6.1).

## Abbreviations

ADAS: Alzheimer Disease Assessment Scale
