# Supplementary material for: The Effect of Transcranial Alternating Current Stimulation With Cognitive Training on Executive Brain Function in Individuals With Dementia: Protocol for a Crossover Randomized Controlled Trial
Source: JMIR Res Protoc. 2022 Apr 27;11(4):e37282. doi: 10.2196/37282 (PMC9096654; doi:10.2196/37282)
Supplement: Multimedia Appendix 1 [file resprot_v11i4e37282_app1.pdf]

Mitacs

December 15, 2021

Application Ref. IT26869

RE: Mitacs Accelerate Proposal

Project Title:	Investigating the Effect of Cognitive Training Simultaneously with Application of either Active or Sham Transcranial Alternating Current Stimulation on the Executive Brain Functions in Dementia Population
Internship Supervisor(s):	Zahra Kazem-Moussavi
Co-Supervisor(s):	Brian Lithgow, Mohammad Jafari-Jozani
Intern(s):	Maria Uehara, Natasha Jacobson, Rashmita Chatterjee, Samaneh Azarbarzin, Sadegh Marzban, TBD
Department:	Department of Electrical and Computer Engineering, Department of Statistics
Institution:	University of Manitoba
Partner Organization:	Riverview Health Centre Foundation, Aster Gardens

Dear applicants,

Your application to Mitacs Accelerate has successfully passed research review. Your research project has been approved for a total grant of $399,999.99 that will be delivered through eligible internships. Your internship should not start prior to confirmation of eligibility as outlined below:

1. Mitacs must receive partner funds before each internship start date to pay the intern(s) on time;
2. Project leads must confirm intern name(s) and start date(s). No costs for an internship can be incurred prior to the research project approval and the receipt of partner funds for the internship;
3. To identify a new intern, the Intern Profile form (including CV and memorandum) must be submitted to Mitacs before the applicable internship's start date and obtain Mitacs approval;
4. Project lead(s) should consult with the appropriate office(s) at their academic institution(s) to determine whether: Research Ethics Board certification(s), permit(s), or approval(s) is/are required to conduct their activities.

All awards are subject to ongoing program eligibility, continuation of funding by the partner organization(s), and continuation of funding by our government partners. Mitacs Accelerate gratefully acknowledges the financial support of the Government of Canada and the Province of Manitoba.

Welcome to Mitacs Accelerate. With this program, interns can apply their specialized expertise while companies gain a competitive advantage. We look forward to the success of your project.

If you have any questions, please contact Stephanie Char, Grant Management Specialist, at schar@mitacs.ca.

Sincerely,

R. Ben Mrad,

Ridha Ben Mrad
Chief Research Officer and Associate Academic Director

Enclosures:

- Appendix A: Referee comments
- Appendix B: Terms and Conditions of the Award
- Attachment: Intern Onboarding Information

Appendix A: Referee Comments

The applicants have satisfactorily addressed the reviewer's concerns. We wish the applicants success in their research.

We expect that you and your collaborating researchers will follow the directives provided by applicable institutional, municipal, provincial/territorial, and federal bodies in relation to the COVID-19 pandemic. For example, mitigation strategies may be required to accommodate requirements for physical distancing and limitations on gathering size.

Appendix B: Mitacs Accelerate - Terms and Conditions of the Award

Please read all conditions carefully. Any questions or requests for clarification should be directed to your Grant Management Specialist listed in the letter above.

1. General guidelines:

1.1.The average amount of partner interaction for each four-month internship across the project should be 50% (or minimum of 25% with sufficient justification in original application).

1.2.The maximum allowable number of Accelerate internships varies depending on the intern's academic designation:

1.2.1. College students can complete three internship unit;
1.2.2. Master's students can complete up to four internship units;
1.2.3. PhD students can complete up to eight internships units; and,
1.2.4. Postdoctoral fellows can complete up to nine internships units.

1.3.The start date of each internship cannot predate the research approval of the project and the receipt of partner funds for the internship.

1.4.Awarded funds must be allocated according to the internship period(s) and stipend/salary allocations, which will be outlined on the Mitacs Award Letter.

1.5.Please report any substantial change (internship dates, project personnel, partner organizations, etc.) concerning your project immediately to your Mitacs Grant Management Specialist contact.

2. Funding:

2.1. Contribution funds from the partner organization must be paid to Mitacs prior to the internship start date to facilitate efficient payment process for interns.

2.2. Funding will not be released for un-named interns.

2.3. The stipend portion of the award must be spent as outlined in the award letter. No extensions can be granted on stipend amounts.

2.4. The research grant portion of the award must be spent by end date of the award. Any remaining research funds may be used by the academic supervisor to supplement the intern's stipend at the Academic Supervisor's discretion. Any funds not spent by this date must be returned to Mitacs unless an extension of the award is granted by Mitacs. To obtain an extension, the supervisor needs to contact Mitacs before the end date of the award, with justification of such a request.

2.5. In-kind contributions cannot be used towards matching Mitacs funds.

2.6. Inter-university transfers are not permitted.

3. Intern Stipends:

3.1. Each intern must receive a minimum $10,000 stipend/salary for each internship unit.

3.2. All stipend/salary funds must be paid over a period of no less than 4 months and no greater than 6 months per internship unit.

4. Research Funds:

4.1. The remaining funding may be used to cover expenses in support of the internship research and its related costs, as outlined on Mitacs's Use of Funds page. https://www.mitacs.ca/en/use-funds

4.2. Relevant research expenses incurred outside of the indicated funding period may be approved by Mitacs on a case-by-case basis.

5. Reporting:

5.1. If the award straddles fiscal years, the University will provide a separate Form 300 (statement of account) for each account by May 31st for each fiscal year until the account reaches a zero balance. The University will provide a Final Form 300 for each account within 30 days of the end date of this award. The Academic Supervisor and Finance Officer must sign the Form 300s and forward to Mitacs once finalized. Please include the Application Reference number and Project Title on the Form 300 and submit to form300@mitacs.ca

5.2. Projects involving more than 50 internship units and spanning longer than 2 years are required to submit a progress report to Mitacs at the mid-point of the project. Please designate one academic supervisor to be the coordinator who is responsible for submitting the report to Mitacs. Failure to submit a satisfactory report may result in delay or cancellation of subsequent funding.

5.3. All parties are required to provide Mitacs with a final report and completed exit surveys at the end of the Mitacs Accelerate internship.

Exit Survey links will be emailed to students after the end of their internship, and to partners and professors after the end of the project.

The link to the Final Report is listed below:
http://www.mitacs.ca/sites/default/files/uploads/page/mitacs_accelerate_final_report_mar2013.zip

6. Acknowledgement of Mitacs:

6.1. All Mitacs-sponsored results must explicitly credit Mitacs. This includes work by professors, post-docs, interns, and research associates. Mitacs funding can be acknowledged in publications using the following statement: "This work was supported by Mitacs through the Mitacs Accelerate Program." All networking activities initiated by your project for which Mitacs funds are used must also credit Mitacs. For advertising purposes, the Mitacs logo is available in several formats for download from the website at www.mitacs.ca
