# Supplementary material for: The Effect of Transcranial Alternating Current Stimulation With Cognitive Training on Executive Brain Function in Individuals With Dementia: Protocol for a Crossover Randomized Controlled Trial
Source: JMIR Res Protoc. 2022 Apr 27;11(4):e37282. doi: 10.2196/37282 (PMC9096654; doi:10.2196/37282)
Supplement: Multimedia Appendix 2 [file resprot_v11i4e37282_app2.pdf]

Mitacs

October 8, 2021

Application Ref. IT26869

RE: Mitacs Accelerate Proposal

Project Title:	Investigating the Effect of Transcranial Alternating Current Stimulation with and without Simultaneous Cognitive Training on the Executive Brain Functions in Dementia Population
Internship Supervisor(s):	Zahra Kazem-Moussavi
Co-Supervisor(s):	Brian Lithgow, Mohammad Jafari-Jozani
Department:	Department of Electrical and Computer Engineering, Department of Statistics
Institution:	University of Manitoba
Partner Organization:	Riverview Health Centre Foundation, Aster Gardens

Dear applicants,

Your recent proposal for a Mitacs Accelerate internship project has been reviewed. Based on this review, we request that you submit a revised proposal addressing the following comments (see Appendix A).

Please address the comments in your revised submission so that we may continue considering your proposal for funding. Please include a cover letter indicating your response to any specific points and highlight any changes to your proposal so they are easily identifiable. We look forward to receiving your revised proposal.

Please note that you have 45 days to respond to the request for revisions. If your revised proposal is received by Mitacs more than 45 days after the date of this letter, it will be treated as a new application.

If you have any questions about this letter, you may contact Stephanie Char, Grant Management Specialist, at schar@mitacs.ca.

Yours truly,

R. Ben Mrad,

Ridha Ben Mrad
Chief Research Officer and Associate Academic Director

Appendix A: Referee Comments

Your application has been reviewed by four external experts. Overall, the reviewers have found the project interesting, ambitious, and appropriate for the academic level of the interns. However, the reviewers have raised concerns about certain aspects of the projects objectives, methodology, and timeline that we would like you to address in a revised version of the proposal.

For your revisions, please focus on addressing the following comments from Reviewer 1 (Question 6), Reviewer 2 (Questions 5 and 6), and Reviewer 3 (Questions 4 and 6).

The full reviewer reports are provided for your consideration.

Reviewer 1

1) Mitacs Accelerate supports research-based internships. Does the proposed project qualify as research in its discipline?
Yes it does. This project aims to assess the efficacy of an intervention (tACS + Cog training) on older adults in a placebo-controlled study. The project also aims at developing predictors of response to the intervention. The investigators plan to use Electrovestibulography (EVestG), Egocentric Spatial Assessments using Virtual Reality Navigation, and fNIRs to follow the impact of the intervention and develop predictors of response to treatment. Two hypotheses are proposed: a) there would be a direct link between the deficiencies in the vestibular system and AD. b) Spatial orientation would be significantly deficient early on in AD. Investigators have pilot data with Active tACs + Cog training. Through this study, they hope to better assess the efficacy of this intervention through a placebo-controlled trial. Investigators also have pilot data using EvestG in dementia with two prior Mitacs Grant. The pilot indicates EvestG features that predict response to rTMS in dementia and correlates with AD severity.

2) Is the project appropriate for the academic degree level of the intern(s)?
Yes. It has a good combination of technical and neuroscientific inquires appropriate for biomedical engineering interns.

3) Are the objectives clear?
Yes. They are clearly stated.

4) Is the methodology appropriate to achieve the objectives?
Yes. The trial design and methodologies are chosen with rigor.

5) Is the timeline realistic?
They wish to study 77 participants in two years. They have a track record in running studies in patients with dementia. So it seems feasible given their access to resources.

6) Other comments and suggestions for the applicants. For example, is there prior work that should be considered by the researchers?
I can think of two minor improvements:

i) In the economic development section it is indicated that "The proposed project will enable the first partner (RHCF) to offer a better and more optimized selection of services for enhancing the quality of life of RHC residents. If rTMS is validated as a useful treatment tool for Alzheimer's disease, RHCF will benefit by having access to this tool for its many residents ..." However, the study is focused on tACS + Cog training. I wonder if "rTMS' is a typo?

ii) It's not immediately clear what patient population they would be studying. It indicated 'older adult' at the beginning. I had to look this up buried in the methodology that the aim is to have older adults with 5

Reviewer 2
1) Mitacs Accelerate supports research-based internships. Does the proposed project qualify as research in its discipline?
Yes, the project contains multiple novel and interesting research questions. These include questions relating to i) the efficacy of the proposed tACS Alzheimer's disease (AD) intervention, ii) to a hypothesized relationship between vestibular system deficits and AD;

iii) to fNIRS-measured haemodynamics activity changes following the tACS intervention; and iv) to egocentric spatial awareness assessment using VR.

2) Is the project appropriate for the academic degree level of the intern(s)?
Yes. The proposal includes 5 sub-projects for 6 total trainees at the levels of MSc, PhD, and PDF. The duties outlined for each trainee have appropriate differences in difficulty and assumed skill levels.

3) Are the objectives clear?
Yes. The objectives, whilst quite long and complex, are spelled out and reiterated several times for clarity.

4) Is the methodology appropriate to achieve the objectives?
Yes.

5) Is the timeline realistic?
The timeline of 28 months is ambitious but achievable. The most challenging aspect of adhering to this will likely be recruitment and data acquisition, as the authors' power analyses have set a minimum sample size of 77. The authors have indicated their confidence in this given past recruitment success etc., which is great s enthusiasm. However this earlier success may be attributable to eg a single particularly energetic individual who is no longer part of the process. The authors should prepare a solid contingency plan to be enacted if recruitment rates at a given early time point are not on track. This may include additional hires and narrowing exclusion criteria.

6) Other comments and suggestions for the applicants. For example, is there prior work that should be considered by the researchers?
I may be missing it but I do not see any description of the tACS protocol (esp. spatial montage) in the proposal, despite a lot of other detailed information. I assume from the text that it will follow exactly that of Mouhssavi et al. Frontiers Aging 2021, which gives the following info:

"Transcranial Alternating Current Stimulation (tACS) We used the Soterix Medication tACS Stimulator (Model: 2001) on the tACS group. The stimulation was applied with a sinusoidal waveform at a frequency of 40Hz and a current amplitude of −0.75 mA to +0.75 mA (1.5 mA p-p). The active electrode was placed over the left dorsolateral prefrontal cortex (DLPFC) and the reference electrode was placed on the contralateral supraorbital area. Stimulation was applied simultaneously with the brain exercises per each session of treatment on participants in tACS + Exr group."

Reviewer 3
1) Mitacs Accelerate supports research-based internships. Does the proposed project qualify as research in its discipline?
Yes.

2) Is the project appropriate for the academic degree level of the intern(s)?
The project describes work for several interns at various training stages. The responsibilities of incoming MSc students appear somewhat ambitious; however, the PI has a large laboratory and a strong track record of training HQP suggesting that necessary supports are in place.

3) Are the objectives clear?
The proposal describes a large project with many objectives. Generally, the listed objectives are clear and well thought out.

4) Is the methodology appropriate to achieve the objectives?
Rationale for the fNIRS component of the project is lacking. Why was it chosen as a tool to potentially use for response prediction? The applicants state: "as we are also always exploring new objective physiological monitoring tools, we will also use three novel technologies for monitoring the physiological changes in study participants before and after the proposed training program." This sounds as though these technologies are being used just for the sake of using new technologies. Stronger rationale is needed.

5) Is the timeline realistic?
Yes. The project is ambitious, but the applicants have experience conducting similar work and have provided a reasonable timeline.

6) Other comments and suggestions for the applicants. For example, is there prior work that should be considered by the researchers?
The project title does not seem to match the study design: "Investigating the Effect of Transcranial Alternating Current Stimulation

(tACS) with and without Simultaneous Cognitive Training on the Executive Brain Functions in Dementia Population." This reads as though the effect of tACS on executive brain function will be explored when it is paired with cognitive training and when it is delivered alone without cognitive training. My understanding from the rest of the proposal is that the study is designed to test the effect of combined tACS and cognitive training relative to cognitive training with no tACS (i.e., sham tACS).

The cognitive training that participants will perform in the RCT is not described beyond volume and frequency. I expect that it involves the MindTriggers App described in their prior work, but this could be more clearly stated.

It is unclear how the applicants derived the expected difference in means of the groups as 20 points on the WMS-IV score. The applicants refer to pilot work but is a 20-point change reflective of the cognitive training effect or a tACS effect? Is the difference to be detected at an immediate post-training time point or at a 1 month follow up as in the pilot work?

It would also be helpful for the applicants to provide more information on the study participant pool. For example, over what time period has this large pool of individuals (>400) been recruited to studies with this group? Does the study group have an existing data base of potential participants? The study is being run at Aster Gardens and RHCF center. How many eligible participants are involved with these facilities now?

The expected deliverables for Objective 1 (page 11) are quite vague. How will this study provide an important decision making strategy with regards to dementia treatment? What additional steps will the research team take to facilitate changes in clinical decision making with the study results?

On page 11 "Partner Interaction" the applicants indicate that at one study location the research sessions will be conducted by partner facility staff, and at another location by study interns. Ideally the approach taken across study locations would be the same and some measure of cognitive training fidelity would be included.

It is not entirely clear how Objective 2 (new spatial orientation game) fits in with the rest of the project. All remaining objectives (3 through 6) are linked back to objective 1 only.

Regarding Objective 2 Partner Interaction, it would be helpful to include a clear plan on how insight and feedback from the partner employees will be obtained. Will feedback be provided simply through informal, undocumented conversations or will interviews /focus groups be conducted?

Considering Objective 3, the application would be strengthened by providing evidence that the research team and intern have the expertise to collect, analyze, and interpret fNIRS data. Additionally, with only the Manitoba participants, will the applicants have enough data to evaluate correlations as planned?

The benefits to the interns are quite repetitive and focus on providing interns with exposure to the length process that is research and development. The application would be strengthened by providing more targeted outcomes for the interns based on training stage and background.

The economic orientation of this project is in its potential to reduce the economic burden of dementia by providing a new treatment approach. However, in this section of the application (2.7), the applicants discuss the validation of rTMS as a treatment tool for Alzheimer's disease. To my understanding, the current project is designed to study the effects of tACS on older adults with dementia, not rTMS and Alzheimer's.

Reviewer 4
1) Mitacs Accelerate supports research-based internships. Does the proposed project qualify as research in its discipline?
yes

2) Is the project appropriate for the academic degree level of the intern(s)?
yes

3) Are the objectives clear?
yes

4) Is the methodology appropriate to achieve the objectives?
yes

5) Is the timeline realistic?
yes

6) Other comments and suggestions for the applicants. For example, is there prior work that should be considered by the researchers?
appropriate
